# Factors Surrounding the Healthcare Transition From Pediatric to Adult Care in 5p- Syndrome: A Survey Among Healthcare Professionals

**DOI:** 10.3389/fped.2022.924343

**Published:** 2022-07-08

**Authors:** Yuko Ishizaki, Mari Matsuo, Kayoko Saito, Yoko Fujihira

**Affiliations:** ^1^Department of Pediatrics, Kansai Medical University, Osaka, Japan; ^2^Institute of Medical Genetics, Tokyo Women’s Medical University, Tokyo, Japan; ^3^5p- Syndrome Association Chamomile, Tokyo, Japan

**Keywords:** 5p- syndrome, healthcare transition, physical disability, intellectual disability, siblings

## Abstract

**Background:**

The 5p- syndrome is associated with intellectual disturbance and physical complications from infancy, and patients continue treatment into adulthood. This study aimed to clarify the factors that facilitate and prevent healthcare transition from pediatric to adult care by conducting a questionnaire survey among medical professionals.

**Subjects:**

The survey included 81 medical professionals nominated by an association of families of 5p- patients in Japan. The questions involved medical care for 5p- syndrome in adulthood, experience of transition, and factors facilitating a patient’s transition. Responses were obtained from 32 participants, with 27 answers eligible for analysis.

**Results:**

The questionnaire items involved physical symptoms and concerns regarding support and welfare prompting consult. The most common physical symptom was constipation. Regarding support and welfare, all participants had an experience of receiving consultation about care for the siblings of patients. Three (11.1%) participants had an experience of transition. Regarding the transition of patients with rare diseases or intellectual disturbance, only four (14.8%) believed that progress was being made in the transition.

**Discussion:**

Only 11% of the respondents experienced the transition of patients with 5p- syndrome. Because it is difficult for highly specialized adult care providers to deal with multidisciplinary complications of 5p- syndrome and information on prognosis and natural history is not known, it is presumed that the transition of 5p- syndrome did not progress. Factors to improve the transition of patients with 5p- syndrome and are likely to be effective for the transition of patients with other rare diseases or intellectual disabilities.

## Introduction

The transition of healthcare from pediatric to adult care in patients with childhood-onset chronic illness can be hindered by factors such as intellectual disturbance, physical complications, consultations with physicians in multiple clinical departments, and inadequate understanding of the disease by physicians in adult care departments. In particular, it is difficult for patients with intellectual disabilities to move to adult centered care (1).

The 5p- syndrome is attributed to a chromosomal abnormality and is associated with developmental retardation. This starts in infancy, but the severe intellectual disturbance and physical complications persist into adulthood (2–5). In 1963, 5p- was first reported as Cri du chat Syndrome by Lejeune et al. (6), and it is currently one of the most common chromosomal deletion syndromes, with an incidence of 1:15,000 to 1:50,000 live births (7). In Japan, Higurashi et al. (8) reported a birth prevalence of 5p- syndrome of 2 out of 21,472 consecutive newborn babies at a large maternity hospital in Tokyo.

The main clinical features of patients with 5p- at birth are low birth weight, microcephaly, round face, and characteristic facial features such as large nasal bridge, hypertelorism, epicanthal holds, low-set ears, micrognathia, and typical cry (2). With age, behavioral problems and linguistic disabilities become prominent (2, 3, 9, 10). The severity and spectrum of clinical features (i.e., phenotype) depend on the size and location of the deletion (i.e., genotypes) (11).

Although the mortality in childhood was about 10% in an early report (12), it has since been improving (2). If no major origin defects or other clinical medical conditions exist, the life expectancy appears to be normal (2). In line with improving prognosis, it is also necessary to consider the healthcare transition of 5p- patients to adult centered care. Since patients with 5p- have various symptoms, it is necessary for them to undergo treatment in multiple clinical departments. Thus, we believe that 5p- syndrome may represent a model of medical care and transition of patients with childhood-onset chronic illness who have physical and intellectual disabilities persisting into adulthood. In this study, we conducted a questionnaire survey of medical professionals who were involved in the medical care of patients with 5p-. This study aimed to clarify the status of the transition of patients with 5p- and the factors contributing to the progression of this transition (especially considering physical disabilities and intellectual delay).

## Methods

### Subjects

The study population included 81 medical professionals who were nominated by the 5p- Syndrome Association Chamomile. Established in 1995, it is the only association of the families of patients with 5p- in Japan. There are around 110 families throughout Japan, with patients ranging from newborns to 30 years old. The activities of the association include exchange meetings, information provision, and peer counseling. All medical professionals nominated by the association have been involved in the medical care of patients with 5p-.

### Methods

The survey included questions about medical care for patients with 5p-, experience regarding the transition of patients with 5p-, factors facilitating the transition of patients with rare diseases or intellectual disturbance from pediatric to adult health care, and free descriptions. The survey questions were prepared by representatives of the 5p- Syndrome Association Chamomile, pediatricians, and specialists of pediatric genetics based on and academic views. The questionnaire was distributed by a controller, filled out anonymously by the subject, then returned to the controller (Appendix).

### Ethics

This research was approved by the Ethics Committee of Kansai Medical University Medical Center (No. 2019129).

## Results

### Characteristics of the Participants

Replies were obtained from 32 medical professionals (return rate; 39.5%), and there were 27 answers eligible for analysis. Of the 27 participants, 23 were doctors and 4 were dentists. Among the respondents, 13 were from the Department of Pediatrics, six were from the Department of Genetics, and eight were from other departments, such as Orthopedics, Ophthalmology, Dentistry, Rehabilitation, Neurosurgery, Psychiatry, Otorhinolaryngology, and Internal Medicine. The length of their clinical experience was 27.6 (standard deviation [SD]: 8.9; range: 12–50 years). In terms of experience, these physicians treated an average of 2.8 (SD: 3.9) patients with 5p-, and 19 performed regular medical examinations for patients with 5p-.

### Medical Care for 5p- Syndrome

Table 1 summarizes the items answered by the respondents regarding physical symptoms and concerns regarding support and welfare which have prompted consult. The most common physical symptoms were constipation, sleep disorders, and feeding difficulties, followed by hyperactivity and dysphagia. Regarding consultations for support and welfare, all participants answered they were consulted by the families of patients with 5p- about care for the siblings of patients. Other notable concerns were regarding prognosis, schooling, employment, and the diagnosis of disability.

**TABLE 1:** Physical symptoms and social support and welfare concerns of patients with 5p- syndrome according to healthcare professionals (*N* < 27, multiple answer).

	N	%		N	%

Physical symptoms			Social support and welfare concerns		
Constipation	11	40.7	Care for siblings	27	100
Sleeping disorder	10	37.0	Prognosis	13	48.1
Difficulties of breastfeeding	9	33.3	Education and employment	12	44.4
Hyperactivity	7	25.9	Certification of disabilities	11	40.7
Difficulties in chewing	7	25.9	Medical certificate	11	40.7
Weight loss	6	22.2	Support for medical costs	10	37.0
Aspiration	6	22.2	Cooperation with other	9	33.3
Self-harm behavior	6	22.2	Medical professionals		
Hypotonia	6	22.2	Recurrence within the family	8	29.6
Hearing sensitivity	4	18.2	Patient group	8	29.6
Irritation	4	18.2	Short stay	6	22.2

Answers for the questions about the cited difficulties in medical treatment included “shortage of clinical experience” (*N* = 9, 33.3%), “lack medical information about 5p-” (*N* = 7, 25.9%), “need to attend multiple clinical departments” (*N* = 6, 22.2%), and “transitions to the adult centered care” (*N* = 4, 14.8%).

About visiting multiple departments, 22 respondents (81.5%) said that their patients visited multiple departments. Among these participants, seven (25.9%) reported that they experienced problems in concurrent care with other departments, specifically regarding “increased burden on the patient” (*N* = 6, 33.3%) and “difficulties in information sharing” (*N* = 2, 7.4%).

The most common clinical departments the patients consult other than pediatrics were Otolaryngology (*N* = 11, 40.7%), Rehabilitation (*N* = 10, 37.0%), Ophthalmology, Orthopedics, Dental and Oral Surgery (*N* = 9, 33.3%), and Genetics (*N* = 4, 14.8%). The other departments were General Internal Medicine, Endocrinology, Pediatric Surgery, Orthopedics, Pediatric Cardiology, Neonatology, and even at an epilepsy center.

### Transition of Adult Patient With 5p- Syndrome and Patients With Rare Diseases or Intellectual Disturbance

Only three participants (11.1%) reported that they had an experience of transition, whereas 24 (88.9%) did not. None of the respondents reported that they rejected the request of transition nor did their patients reject the transition request.

Regarding the transition of patients with rare diseases or intellectual disturbance, 4 out of 27 participants (14.8%) believed that progress was being made in the aspect of transition care.

When asked about the factors facilitating the smooth transition of patients with rare diseases or disabilities (multiple answers were allowed), 23 physicians (85.1%) answered “provides a summary of information about the disease together,” “obtains an informal consent in advance from the physician to whom the patient is to be referred,” and “maintains close contact with the physician to whom the patient was referred and accepts referrals.” The other common answers were “health care transition from pediatric to adult care is explained to family members” (*N* = 22, 81.5%), “makes a list of information about medical institutions accepting the transition” (*N* = 18, 66.8%), and “provides detailed information about past medical history” (N = 16; 59.3%) (Table 2). To solve these problems, participants gave the following answers in the free description portion of the questionnaire: “adult physicians provide comprehensive medical care, like pediatricians,” “use of care management guidelines for the relevant disease,” “convincing adult physicians of advantages of transition and disadvantages of continued care by pediatricians,” “collaboration between pediatric and adult medical institutions,” and “making a list of information about medical institutions accepting the transition.”

**TABLE 2:** Factors facilitating the smooth transition of patients with rare diseases or disabilities (*N* = 27, multiple answer).

	N	%
Provides a summary of information about the disease together	23	85.1
Obtains an informal consent in advance from the physician to whom the patient is to be referred	23	85.1
Maintains close contact with the physician to whom the patient was referred and accepts referrals	23	85.1
Healthcare transition from pediatric to adult care is explained to family members	22	81.5
Makes a list of information about medical institutions accepting the transition	18	66.7
Provides detailed information about past medical history	16	59.3

When asked about considerations for patients with rare diseases or disabilities receiving medical care in the community, participants cited the following: “systems for collaboration among medical professionals,” “improvements of regional medical networks,” “having a summary of medical history and test results,” “information sharing in advance, such as susceptibility to infection, means to exercise, and any required medical care,” “willingness of accepting and dealing with difficulties in medical care for patients with intellectual disturbance,” “availability of paramedical staff capable of dealing with patients with intellectual disabilities,” “availability of human resources (divisions) to comprehensively coordinate issues including those related to medical care, welfare, education, and employment,” “considerations such as shortened waiting time and space to wait for medical examinations,” “flexibility to adjust for characteristics of patients/patient families,” and “information sharing at patient/family meetings.”

## Discussion

We administered a questionnaire survey among healthcare professionals to clarify the status of the transition of 5p- syndrome from childhood to adult care, as well as the factors contributing to its progression, especially considering physical disabilities and intellectual delay. Notably, out of 27 respondents, more than 66% reported that patients with 5p- syndrome visited multiple clinical departments, 11% experienced the transition of patients with 5p- syndrome from childhood to adult care, and none reported that they or their patients rejected the transition.

As an interpretation of this result, we considered that 11% of the participants had an experience of complete transition. The remaining 89% were actually treating adult patients with 5p- syndrome. Among professionals whose patients were in childhood and adulthood, some of them were looking for a transition goal but had not yet found it or they had tried to move to adulthood but had not succeeded in it and were trying again. It is also thought that there were some participants who did not feel the need for transition to adult care and thought that there was no problem in continuing to see the adult patients in pediatric clinics. At the time of this survey, the eldest patient was in their 30s, and there were no elderly patients in the 5p- Syndrome Association Chamomile in Japan. As a result, majority of the patients were still being assessed in pediatrics and may have had little experience with transitions.

Regarding medical care for patients with 5p-, participants cited the presence of various physical and behavioral symptoms such as constipation, sleeping disorder, difficulties of breastfeeding, hyperactivity, and difficulties in chewing, suggesting that patients with 5p- continuously need different aspects of medical care. Regarding social support and welfare, the families of patients most commonly consulted about caring for the siblings of 5p- patients, followed by prognosis, employment, and certification of disabilities.

Mental health problems have been reported in the siblings of children with rare diseases (13), chronic illness (14), and developmental disability (15–17). Hodapp et al. (18), examined the stress-related concerns and responses of families of patients with 5p-; it was found that parents and siblings disagreed on the extent of the siblings’ interpersonal concerns. Parents reported that the siblings felt ignored and misunderstood, whereas the siblings themselves rated these concerns at much lower levels. Although this paper was published in the 1990s, sibling care remains a very important issue for the families of patents with 5p-.

Moreover, more than 80% of respondents stated that the transition care of patients with rare diseases or disabilities was not moving forward. These findings are in agreement with those in previous reports regarding the transition of neurological disease patients with intellectual disturbances.

Based on the results of this study, most patients with 5p- syndrome have not transitioned to adult centered care. Since none of the participants reported rejecting/being rejected for transition, it is presumed that no attempt has been made. The reasons may be complex and the following points might be specific to 5p-: (1) 5p- syndrome has multidisciplinary complications, such as cardiovascular, urogenital, musculoskeletal, otolaryngology, ophthalmology, and dentistry, and often requires surgical treatment. Because adult care providers are often specialized and they rarely provide comprehensive medical care like pediatricians, they were not able to handle multidisciplinary complications; (2) Since patients are not accustomed with adult care providers, they do not report their symptoms by themselves due to intellectual disabilities, like those in patients with 5p-; and 3) Little information on prognosis and natural history has been accumulated due to the low incidence of 5p- syndrome.

Not only is 5p- syndrome itself a combination of multiple physical complications and intellectual disability, but the general public also lacks information about the disease. Therefore, disseminating knowledge and social support for this disease may be an effective intervention for improving the rates of transition care. The 5p- Syndrome Association Chamomile, which planned this survey, is promoting a guidebook for patients’ families and medical professionals. Such activities may promote the health care transition of patients with 5p-.

The current status of transition for patients with 5p- can be improved by factors such as collaboration between pediatric and adult departments, information sharing, preparation of medical institution lists, disease guidelines, clarification of the benefits of transition to adult departments, and the availability of human resources to coordinate issues comprehensively. Similarly, these can likely be effective as well for the transition of patients with other rare diseases or intellectual disabilities.

## Conclusion

Because patients with 5p- can be a representative model for patients with physical and intellectual disturbance, the factors which can improve the transition to adult care can similarly be effective as well for patients with other rare diseases or intellectual disabilities.

## Data Availability Statement

The raw data supporting the conclusions of this article will be made available by the authors, without undue reservation.

## Ethics Statement

The studies involving human participants were reviewed and approved by The Ethics Committee of Kansai Medical University Medical Center. Written informed consent for participation was not required for this study in accordance with the national legislation and the institutional requirements.

## Author Contributions

YI, MM, KS, and YF were performed the data collection. YI and MM performed the first draft of the manuscript. KS and YF revised it critically for important intellectual content. All authors contributed to the study conception and design, commented on previous versions of the manuscript, and read and approved the final manuscript.

## Conflict of Interest

The authors declare that the research was conducted in the absence of any commercial or financial relationships that could be construed as a potential conflict of interest.

## Publisher’s Note

All claims expressed in this article are solely those of the authors and do not necessarily represent those of their affiliated organizations, or those of the publisher, the editors and the reviewers. Any product that may be evaluated in this article, or claim that may be made by its manufacturer, is not guaranteed or endorsed by the publisher.

## Appendix

Questionnaire regarding the current status of healthcare transition for the patients with 5p- syndrome

(1). Specialty and experience

 (1) Specialty

 (2) Years of experience

(2). How many patients with 5p- syndrome have you seen in the past? (Yes/No)

 (1) Number of patients seen so far

 (2) Number of patients seen within the last year

(3). Do you see patients with 5p- syndrome on a regular/irregular basis? (Yes/No)

 (1) Regular basis

 (2) Irregular basis

(4). Symptoms of patients with 5p- syndrome

 (1) Please tell us the symptoms of the patients with 5p-syndrome at the time of consultation.

 (a). Constipation (b). Sleep disorders (c). Hearing sensitivity (d). Obesity (e). Weight loss (f). Irritation (g). Aspiration (h). Choking (i). Difficulties in chewing (j). Difficulties in breastfeeding (k). Hand tremor (l). Visual impairment (m). Hearing impairment (n). Hyperactivity (o). Impulsivity (p). Autistic tendency (q). Self-harm behavior (r). White hair (s). Regression (t). Hypotonia (u). Hypertonia (v). Early tooth loss (x). Dysmenorrhea

 (2) Please tell us the support or welfare concerns of the patients with 5p-syndrome

 (a). Prognosis (b). Recurrence within the families (c). Certificate of disabilities (d). Medical certificate (e). Patient groups (f). Education (g). Employment (h). Respite care (i). Short stay (j). Admission to a medical facilities (k). Group home (l). Medical certificate (m). Care for siblings (n). Pension (o). After the death of parents (p). Cooperation with other clinical departments and medical institutions (q). Others

(5). What difficulties do you face in treating patients with 5p- syndrome?

 (a). Nothing in particular (b). Lack of medical information about 5p- syndrome (c). Shortage of clinical experience (d). Not enough time for consultation (e). Frequent visits (f). Many kinds of prescription (g). Various complications (h). Needs of long-term management (i). Need to attend multiple clinical departments (j). Cooperation cannot be obtained from patients due to intellectual delay (k). Difficulties in communication with patients (l). Multiple uses of social welfare (m). Preparing lots of documents (n). Transition to adult centered care (o). Others

(6). Does your patient with 5p- syndrome see other departments? (Yes/No)

(7). Did you ever face any issues with other medical departments? Please describe

 (a). Difficulties in information sharing

 (b). Treatment policy is affected

 (c). Difficult to know the prescription of the other clinical department

 (d). The necessary inspection is omitted

 (e). Increasing medical examination

 (f). Increase burden of the patients

(8). Have you ever experienced a transition from pediatric to adult health care for your patients with 5p- syndrome? (Yes/No)

(9). Have you ever been denied transition of the patients with 5p- syndrome? (Yes/No)

(10). Do you think the transition of patients with rare diseases and disabilities is progressing? (Yes/No)

(11). What are the factors that facilitate the smooth transition of patients with rare diseases and disabilities?

 (a). Provides a summary of information about the disease together

 (b). Obtains an informal consent in advance from the physician to whom the patient is to be referred

 (c). Maintains close contact with the physician to whom the patient was referred and accepts referrals

 (d). Healthcare transition from pediatric to adult care is explained to family members

 (e). Makes a list of information about medical institutions accepting the transition

 (f). Provides a detailed information about past medical history

(12). What kind of consideration do you think patients with rare diseases or lifelong diseases need to take when receiving medical care in the community? (free description)
